# KARAJ: An Efficient Adaptive Multi-Processor Tool to Streamline Genomic and Transcriptomic Sequence Data Acquisition

**DOI:** 10.3390/ijms232214418

**Published:** 2022-11-20

**Authors:** Mahdieh Labani, Amin Beheshti, Nigel H. Lovell, Hamid Alinejad-Rokny, Ali Afrasiabi

**Affiliations:** 1 Biomedical Machine Learning Lab, The Graduate School of Biomedical Engineering, University of New South Wales (UNSW), Sydney, NSW 2052, Australia; 2 Data Analytics Lab, Department of Computing, Macquarie University, Sydney, NSW 2109, Australia; 3 The Graduate School of Biomedical Engineering (GSBmE), University of New South Wales (UNSW), Sydney, NSW 2052, Australia; 4 Tyree Institute of Health Engineering (IHealthE), University of New South Wales (UNSW), Sydney, NSW 2052, Australia; 5 UNSW Data Science Hub, University of New South Wales (UNSW), Sydney, NSW 2052, Australia; 6 Health Data Analytics Program, Centre for Applied Artificial Intelligence, Macquarie University, Sydney, NSW 2109, Australia; 7 Centre for Immunology and Allergy Research, Westmead Institute for Medical Research, University of Sydney, Sydney, NSW 2006, Australia

**Keywords:** biological data, Genomics, transcriptomics, Download, Bioinformatics, sequence data, FASTQ, Linux

## Abstract

Here we developed *KARAJ*, a fast and flexible Linux command-line tool to automate the end-to-end process of querying and downloading a wide range of genomic and transcriptomic sequence data types. The input to KARAJ is a list of PMCIDs or publication URLs or various types of accession numbers to automate four tasks as follows; firstly, it provides a summary list of accessible datasets generated by or used in these scientific articles, enabling users to select appropriate datasets; secondly, *KARAJ* calculates the size of files that users want to download and confirms the availability of adequate space on the local disk; thirdly, it generates a metadata table containing sample information and the experimental design of the corresponding study; and lastly, it enables users to download supplementary data tables attached to publications. Further, *KARAJ* provides a parallel downloading framework powered by *Aspera connect* which reduces the downloading time significantly.

## 1. Introduction

Nowadays, with the advent of new biomedical technologies, the growth rate of genomic and transcriptomic data has been unprecedented (~40 billion gigabytes of new data every year) [1]. This has allowed researchers to access invaluable data sources for performing computational and statistical analyses to develop a list of testable hypotheses focused on decoding the information of genetic materials [2,3]. This process via identifying a list of genetic patterns and molecular signatures, can lead to a better understanding of molecular mechanisms underlying biological processes with the goals of improving human health, developing preventive measures, and curing complex diseases [2,3]. A primary bottleneck in achieving this aim is the lack of quick and convenient access to genomic/transcriptomic data, particularly those that are highly relevant to the desired research question. These data are commonly stored in FASTQ, FASTA, BAM, SAM, GFF, GTF, and VCF file formats, referred to as sequence data [4,5,6,7,8]. Multiple databases including Sequence Read Archive (SRA) [9], Gene Expression Omnibus (GEO) [10,11], European Molecular Biology Lab-European Bioinformatics Institute European Nucleotide Archive (EMBL-EBI ENA) [12], DNA Data Bank of Japan Gene Expression Archive (DDBJ GEA) [13], and Encyclopedia of DNA Elements (ENCODE) database [14,15] support storage and access to sequence data through a unique accession number to each study and experiment.

The architecture of data storage systems in the aforementioned databases and the connection between these databases have been reviewed by Gálvez-Merchán, et al. in more detail [16]. Although existing tools such as *ffq* [16], *SRA Toolkit* [9], *Pysradb* [17], *SRA-explorer* [18] and *nf-core/fetchngs* [19] facilitate access to genomic/transcriptomic sequence data, there are practical limitations that need to be addressed either manually or with extra in-house scripting. This makes the process of querying and downloading these data time consuming and technically challenging.

The first challenge is finding accession numbers; researchers need to manually check several research articles individually to find the accession numbers of genomic/transcriptomic data relevant to their research questions, a time-consuming and tedious task. Currently, there are tools to ease this step [9,11,17,20,21,22,23,24], but they are not automated and powerful enough, and still there are some steps (going through the text of multiple papers and searching for sequence data IDs) that need to be performed manually. The limitations of these tools have been reviewed elsewhere [25].

The second challenge is that these files are customarily extremely large, causing a significant impediment before beginning the desired analysis. There are options available for rapid downloading of sequence data. The most efficient available option to download sequence data is *IBM Aspera connect* (*Aspera*) which uses parallel transferring to accelerate the downloading process [26]. Our in-house experiment with downloading ten FASTQ files obtained from GSE126379 [27] indicates that *Aspera* is 3.2, 3.1 and 5.0 times faster than *wget*, *curl*, *fastq-dump*, respectively. Downloading via *Aspera* protocol requires a specific type of URL, which consists of a public key authentication and a resource path of corresponding data to a network-optimized data transfer protocol (FASP—Fast Adaptive and Secure Protocol). The *ffq* tool acquires the download link for these files using accession numbers; however, it does not generate the *Aspera* download link [16]. *SRA-explorer* retrieves the download links for both the transfer protocols FTP and *Aspera*, but it must be downloaded manually from its webpage, followed by in-house scripting to execute the downloading process [18]. *SRA Toolkit* [9] also retrieves FTP protocol links via SRA accession numbers; however, its downloader *fastq-dump* is not an efficient option compared to *Aspera* as it is less stable and also slower (showed by our in-house experiment). The *Pysradb* tool [17] is another available option that retrieves *Aspera* protocol download links for different types of accession numbers. However, it does not support all types of accession numbers including BioProject database identifier (PRJNA) accession number type.

The third challenge arises when a user needs to analyze many files in the local system, which is usually limited in memory capacity. Because the amount of space needed to store these files is unknown and there is no straightforward way to determine this and the memory storage needed on the local drive, users are not able to acknowledge space allocation. Therefore, it is a common experience for researchers to attempt to download all files of interest and then encounter insufficient local storage space, resulting in the killing of the process and requiring the whole process to be redone. None of the above-mentioned tools offer any solution for this issue.

Lastly, to the best of our knowledge, there is no currently available tool for retrieving processed data of studies published as supplementary tables attached to scientific articles. Commonly, these supplementary data tables can be crucial for downstream analyses of sequence data.

In summary, with the exponential growth rate of new genomic datasets, current pipelines need to be improved by being end-to-end automated. To address this unmet need, we developed a flexible and user-friendly command-line tool *KARAJ* to automate and streamline querying and downloading sequence data. This automation makes performing genomic data analyses more efficient.

## 2. Description

**Figure 1:**
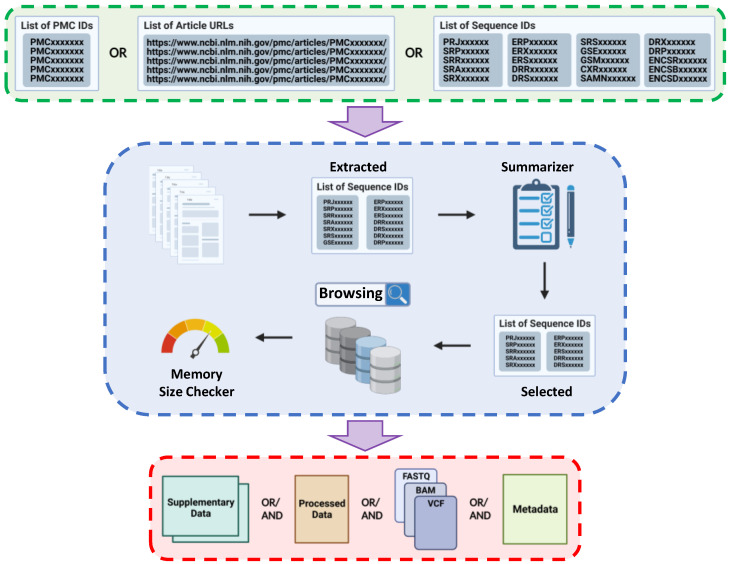
The architecture of *KARAJ*. Input and output file formats are shown by green and red boxes, respectively. The blue box represents the processing steps provided by *KARAJ*. The input to *KARAJ* is a list of either PubMed Central PMCIDs or URLs for articles. *KARAJ* then mines the text of corresponding articles for the accession numbers (Extracted list). Then, *KARAJ* generates a report summary of these accession numbers containing the information including number of samples, description, experimental design, and the sequencing technology. This report summary gives the user the opportunity to choose accession numbers that are of interest (Selected list). *KARAJ* fetches the header for data linked to these accession numbers and calculates the size of these data and checks with the local drive to ensure the availability of adequate space. When adequate local storage space exists, *KARAJ* downloads all files using a parallel framework powered by the *Aspera* protocol. *KARAJ* also accepts list of accession numbers as an input to retrieve sequence data. Image created with BioRender.com under the *NX24GYLITA* agreement number.

*KARAJ* provides an end-to-end automated platform for querying and downloading a wide range of biological data types. *KARAJ*: (i) provides a summary list of accessible datasets generated by, or used in, scientific articles and enables the user to select datasets they want to download; (ii) calculates the size of the selected datasets and confirms availability of adequate local storage space; (iii) generates a metadata table containing sample information and experimental design of the corresponding study; (iv) enables users to download supplementary data tables attached to publications; and (v) supports PRJNA ID to fetch genomic and transcriptomic data (Figure 1).

*KARAJ* takes advantage of the Lynx package [28] to mine the text of research articles for accession numbers and Supplementary Materials. For mining purposes, *KARAJ* supports both PubMed Central unique reference number (PMCID) and publication URL. *KARAJ* utilizes *ffq* [16] to retrieve the download links for various types of accession numbers for databases including SRA, GEO, DDBJ, ENA, and ENCODE. Using *Entrez Direct* [21], *KARAJ* converts PRJNA ID to SRP ID and then fetches the download link using *ffq* [16] through SRP ID for the corresponding PRJNA ID. *KARAJ* downloads selected accession numbers and Supplementary Materials by the user through *Aspera* [26] and *axel* [29] packages, which are known for rapid downloading (Figure 1). Lastly, *KARAJ* provides a parallel framework for running these packages which speeds up the downloading process by at least two times and up to the number of local system cores. We evaluated the *KARAJ* parallel framework for *Aspera*, and it reduced the downloading time of GSE126379 sequence data by 3.6-fold using 8 cores compared to *Aspera* alone.

## 3. Installation

KARAJ is implemented in bash as two shell scripts, Installer and KARAJ tool. The Installer script checks the availability of seven packages required for executing KARAJ, which are ffq [16], pysradb [17], Lynx [28], IBM Aspera connect [26], axel [29], wget [30] and Entrez Direct [21] and installs these dependencies if needed. KARAJ is flexible in using the number of cores on the local system, by a default setting, it automatically recognizes the number of available cores on the local system and uses n-1 number of cores for execution. The user can override this by including the appropriate command line option. The KARAJ tool and its related instructions are provided at https://github.com/GTP-programmers/KARAJ (accessed on 18 November 2022).

## 4. Tutorial

Below is a list of operations that are supported by KARAJ. A summary of options and common errors is provided in Supplementary Tables S1 and S2.

$ ./KARAJ.sh -l URL1 URL2 URL3

The URL(s) of the article(s) that the user is willing to mine for accession numbers is (are) given with the -l option. More than one URL can be specified by separating each URL using a space. The URL(s) must be for the full text version of the research article(s). There is no limit to the number of URLs.

$ ./KARAJ.sh -p PMCID1 PMCID2 PMCID3

The option -p corresponds to PMCID(s) of article(s). Specifying more than one PMCID is possible by separating each PMCID using a space. Users can specify as many PMCIDs as they wish.

$ ./KARAJ.sh -i accession1 accession2 accession3

The option -i corresponds to accession number(s). Using this option, the user can download sequence data linked to that accession number. Multiple accession numbers can be passed to this option. Karaj supports a wide range of types of accession numbers. Given that KARAJ is powered by ffq [16], it supports PRJNA, SRP, ERP, GSE, SRR, SRA, SRX, SRS, ERX, ERS, ERP, DRR, DRS, DRX, DRP, GSM, ENCSR, ENCSB, ENCSD, CXR and SAMN.

$ ./KARAJ.sh -f [1/2/3]

The list of URLs, PMCIDs or accession numbers can be passed to KARAJ as a file with the option -f. The value 1 corresponds to a file named “PMCIDS.txt” in the working directory containing a list of URLs for several articles. The value 2 corresponds to a file named “ACCESSIONS.txt” in the working directory containing a list of accession numbers. The value 3 corresponds to a file named “URLS.txt” in the working directory containing a list of URLs for several articles. Each line in “PMCIDS.txt”, “URLS.txt” and “ACCESSIONS.txt” must contain only one entity.

$ ./KARAJ.sh -t [bam/vcf/fasta/fastq]

With the -t option, the user can filter the selected accession numbers for those with specific file formats (bam, vcf, fasta or fastq). By default, KARAJ downloads all datasets corresponding to the passed accession numbers. This option must be used along with one of the options -p, -l or -f.

$ ./KARAJ.sh -o /directory/output

The directory to save downloaded datasets can be specified using the -o option. By default, the current working directory is designated to save downloaded datasets.

$ ./KARAJ.sh -s [0/1]

The Supplementary Data attached to the articles can be obtained using the -s option. By passing 1, only supplementary tables will be downloaded. The value 0 for this option ignores downloading supplementary tables. This option must be used along with one of the options -p, -l or -f.

$ ./KARAJ.sh -u

This option prints the usage instruction and examples.

$ ./KARAJ.sh -m [0/1]

Using this option, the metadata of selected accession numbers can be retrieved. By passing 1, only (not any other data linked to the corresponding accession numbers) the metadata table will be downloaded. The value 0 for this option ignores downloading metadata tables. This option must be used along with one of the options -p, -l or -f.

$ ./KARAJ.sh -n [0/1]

Using this option, the processed data of selected accession numbers can be retrieved. By passing 1, only the processed data will be downloaded. The default value is 0, which ignores downloading processed data. This option must be used along with one of the options -p, -l or -f.

$ ./KARAJ.sh -h

This option prints the list of all available options of KARAJ tool.

## 5. Usage Examples

The practical application of KARAJ in automation and streamlining the process of querying and downloading various types of sequence data files has been assessed using the following case studies.

### 5.1. Scenario 1

We evaluated the performance of KARAJ in rapid downloading transcriptomic sequence data through accession numbers published by multiple number of articles [27,31,32,33,34]. KARAJ saves these files in separate directories named by the accession numbers for ease of performing downstream analyses. KARAJ also generates a summary table named info.txt containing PubMed URL, Title, Abstract, accession numbers used/published and PMID for article(s) corresponding to PMCID(s)/URL(s) of articles passed to KARAJ.

Command for downloading sequence data of one accession number:

$ ./KARAJ.sh -i GSE126379

Command for downloading sequence data of multiple accession numbers:

$ ./KARAJ.sh -i GSE126379 GSE92521 PRJNA427709 SRR10668798 GSE115469

Command for downloading sequence data of a list of accession numbers:

First, make a file in the working directory entitled “ACCESSIONS.txt” containing the list of accession numbers. Then, run the following command.

$ ./KARAJ.sh -f 1

### 5.2. Scenario 2

Using KARAJ, we mined the text of several scientific articles [27,34,35,36,37,38,39,40,41,42,43] for accession numbers using PMCID of these articles. KARAJ saves downloaded files in separate directories named by the accession numbers for ease of performing downstream analyses. Passing value 0 to the option -s, halts downloading supplementary tables attached to the article(s) corresponding to the passed PMCID(s).

Command for mining the text of an article for accession numbers and downloading sequence data corresponding to them—using PMCID of the article (see Supplementary Figure S1):

$ ./KARAJ.sh -p PMC6492329 -s 0

Command for mining the text of multiple articles for accession numbers and downloading the sequence data corresponding to them—using PMCID of the articles (see Supplementary Figure S2):

$ ./KARAJ.sh -p PMC7182534 PMC6492329 PMC8000127 PMC6957475 PMC8455923 PMC8844275 PMC8426200 PMC7789210 -s 0

Command for mining a list of articles for accession numbers and downloading the sequence data corresponding to them—using PMCID of the articles:

First, make a file in the working directory entitled “PMCIDS.txt” containing the list of article PMCIDs. Then, run the following command.

$ ./KARAJ.sh -f 2 -s 0

### 5.3. Scenario 3

Using KARAJ, we mined the text of several scientific articles [27,35,36,37] for accession numbers using URL of these articles. KARAJ saves downloaded files in separate directories named by the accession numbers for ease of performing downstream analyses. Passing value 0 to the option -s, halts downloading supplementary tables attached to the article(s) respective to the passed URL(s).

Command for mining the text of an article for accession numbers and downloading sequence data corresponding to them—using URL of the article:

$ ./KARAJ.sh -l https://www.ncbi.nlm.nih.gov/pmc/articles/PMC6492329/ (accessed on 12 August 2022) -s 0

Command for mining the text of multiple articles for accession numbers and downloading the sequence data corresponding to them—using URL of the articles:

$ ./KARAJ.sh -l https://www.ncbi.nlm.nih.gov/pmc/articles/PMC6492329/ (accessed on 12 August 2022) https://www.ncbi.nlm.nih.gov/pmc/articles/PMC7182534/ (accessed on 12 August 2022) -s 0

Command for mining the text of a list of articles for accession numbers and downloading the sequence data corresponding to them—using the article URLs: First, make a file in the working directory entitled “URLS.txt” containing the list of article URLs. Then, run the following command.

$ ./KARAJ.sh -f 3 -s 0

### 5.4. Scenario 4

We here show a practical example of using KARAJ in retrieving the supplementary tables attached to several articles [27,34,35,37,38,39,40,41,42] using the PMCID and URL of these articles. KARAJ saves retrieved the supplementary tables in separate directories named by the PMCID.

Command for downloading supplementary tables using article URL:

$ ./KARAJ_V1.sh -l https://www.ncbi.nlm.nih.gov/pmc/articles/PMC6492329/ (accessed on 12 August 2022) -s 1

Command for downloading supplementary tables using article PMCID (see Supplementary Figure S3):

$ ./KARAJ.sh -p PMC6492329 -s 1

Command for downloading supplementary tables of multiple articles using PMCID:

$ ./KARAJ.sh -p PMC7182534 PMC6492329 PMC8000127 PMC6957475 PMC8455923 PMC8844275 PMC8426200 PMC7789210 -s 1

Command for downloading supplementary tables of a list of articles using PMCID:

First, make a text file in the working directory entitled “PMCIDS.txt” containing the list of article PMCIDs. Then, run the following command.

$ ./KARAJ.sh -f 2 -s 1

Command for downloading supplementary tables of a list of articles using article URL:

First, make a file in the working directory entitled “URLS.txt” containing the list of article URLs. Then, run the following command.

$ ./KARAJ.sh -f 3 -s 1

### 5.5. Scenario 5

We evaluated the performance of the memory check module of KARAJ using data generated by MacParland, et al. [36]. Since the size of this sequence data (277 GB) is larger than the free space available in the local disk (7.6 GB), KARAJ halts the downloading process and prints an error of memory size limitation (see Supplementary Figure S4).

$ ./KARAJ.sh -p PMC6197289 -s 0

### 5.6. Scenario 6

Here, we retrieved the metadata table for sequence data of the GSE126379 accession number. KARAJ saves metadata tables in separate directories named by the respective accession numbers.

$ ./KARAJ.sh -i GSE126379 -s 0 -m 1

## 6. Conclusions

*KARAJ* provides a much-needed user-friendly framework to automate and streamline genomic and transcriptomic sequence data downloads. *KARAJ* allows users to automatically search for accession numbers in a list of research articles and rapidly download sequence data corresponding to these accession numbers. In addition, *KARAJ* allows users to retrieve processed data published as supplementary tables attached to research articles automatically. *KARAJ* reduces the querying and downloading time greatly due to its parallel downloading framework powered by Aspera, which speeds up the process of analyzing raw genomic data as well as downstream analyses. In addition, these superior features allow the computational resources allocated to bioinformatics laboratories to be used more efficiently.

## Data Availability

The data underlying this article are available at the Supplementary Materials. The *KARAJ* tool is also publicly available on https://github.com/GTP-programmers/KARAJ (accessed on 18 November 2022).

## Supplementary Materials

The following supporting information can be downloaded at: https://www.mdpi.com/article/10.3390/ijms232214418/s1.
