# Supplementary material for: KARAJ: An Efficient Adaptive Multi-Processor Tool to Streamline Genomic and Transcriptomic Sequence Data Acquisition
Source: Int J Mol Sci. 2022 Nov 20;23(22):14418. doi: 10.3390/ijms232214418 (PMC9694301; doi:10.3390/ijms232214418)
Supplement: Supplementary file 1 [file ijms-23-14418-s001.zip › ijms-1889594-supplementary.pdf]

Supplementary Table S1: The list of common error messages.

Error message	Description
"Invalid option, please check help using -h option"	The error message states that a wrong option is passed.
"Value is missing for option [X]"	The error message states that the value is missed for a particular option. X corresponds to any options.
"One of the obligatory options is missing"	The error message states that one of the options -p, -l, -f or -i has not passed.
"Conciliatory options have passed"	One of the options -p, -l or -f can be passed at the time.
"No sample found. Either the provided accession number is invalid or raw data was not provided for this record"	The error message states that provided accession number is not registered on the respective database.
"The needed memory size for downloading specified datasets is larger than the free space available on the designated local directory. Please change the directory."	The error message states that there is no adequate space on the designated directory to download all specified datasets.

Supplementary Table S2: The list of options and operations.

Options	Description	Default	Syntax
-l	Passing URL(s)	empty	`./KARAJ.sh -l [URL1 URL2 ... URLn]`
-p	Passing PMCID(s)	empty	`./KARAJ.sh -p [PMCID1 PMCID2 ... PMCIDn]`
-o	Output working directory	The current working directory	`./KARAJ.sh -o [directory/output]`
-t	Specifying type of files formats	KARAJ downloads all of file types	`./KARAJ.sh -t [bam/vcf/fastq]`
-s	Downloading supplementary tables. KARAJ does not download supplementary data tables	0	`./KARAJ.sh -l [0/1]`
-f	Passing list of URL(s), PMCID(s) or accession number(s). Value 1 corresponds to a file named “PMCIDs” for passing a list of PMCIDs. Value 2 corresponds to a file named “ACCESSIONS” for passing a list of accession numbers. Value 3 corresponds to a file named “URLS” for passing a list of URL(s). These files must be created in the working directory. Each line in either “PMCIDS”, “URLS” and “ACCESIONS” must contain only one entity.	empty	`./KARAJ.sh -f [1/2/3]`
-i	Passing accession number(s)	empty	`./KARAJ.sh -i [SRR/SRP/PRJ/PRJNA]`
-d	Selecting accession number(s) to download. Value 1 corresponds to go to selection module before downloading files, (0): downloads all files. This option must be passed along with options -l, -p, -f or -i.	KARAJ downloads all files.	`./KARAJ.sh -d [1/0]`
-m	Downloading metadata. This option must be passed along with options -l, -p, -f or -i.	empty	`./KARAJ.sh -m [URL(s)]?`
-h	Help	empty	`./KARAJ.sh -h`
-u	Usage and examples	empty	`./KARAJ.sh -l [URL(s)]`
-c	Number of cores	Number of accessible cores minus one	`./KARAJ.sh -c [core]`
