# Supplementary figures and images for: KARAJ: An Efficient Adaptive Multi-Processor Tool to Streamline Genomic and Transcriptomic Sequence Data Acquisition
Source: Int J Mol Sci. 2022 Nov 20;23(22):14418. doi: 10.3390/ijms232214418 (PMC9694301; doi:10.3390/ijms232214418)

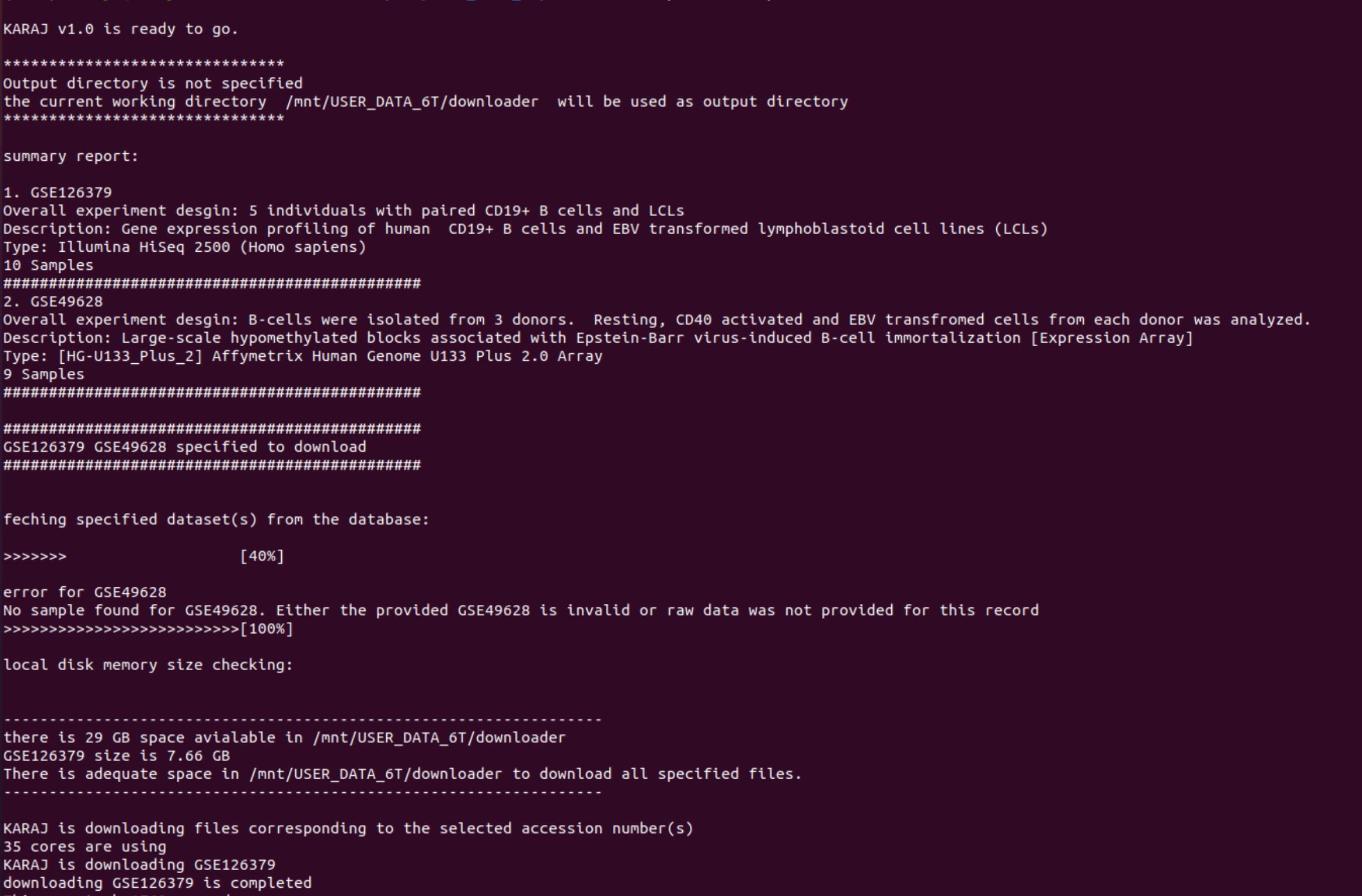

Supplement: Supplementary file 1 [file ijms-23-14418-s001.zip › Supplementary_Figure S1.JPG]

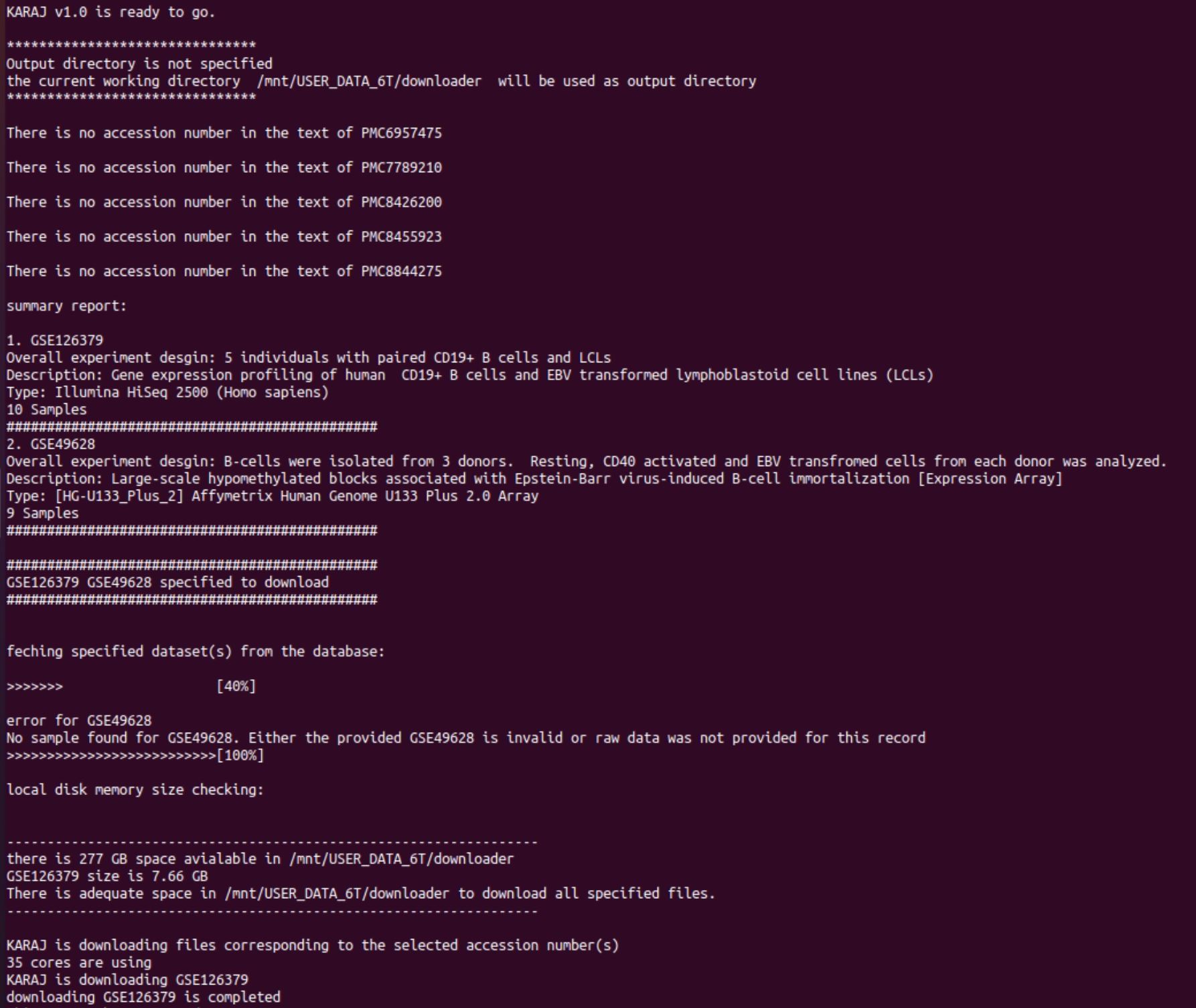

Supplement: Supplementary file 1 [file ijms-23-14418-s001.zip › Supplementary_Figure S2.JPG]

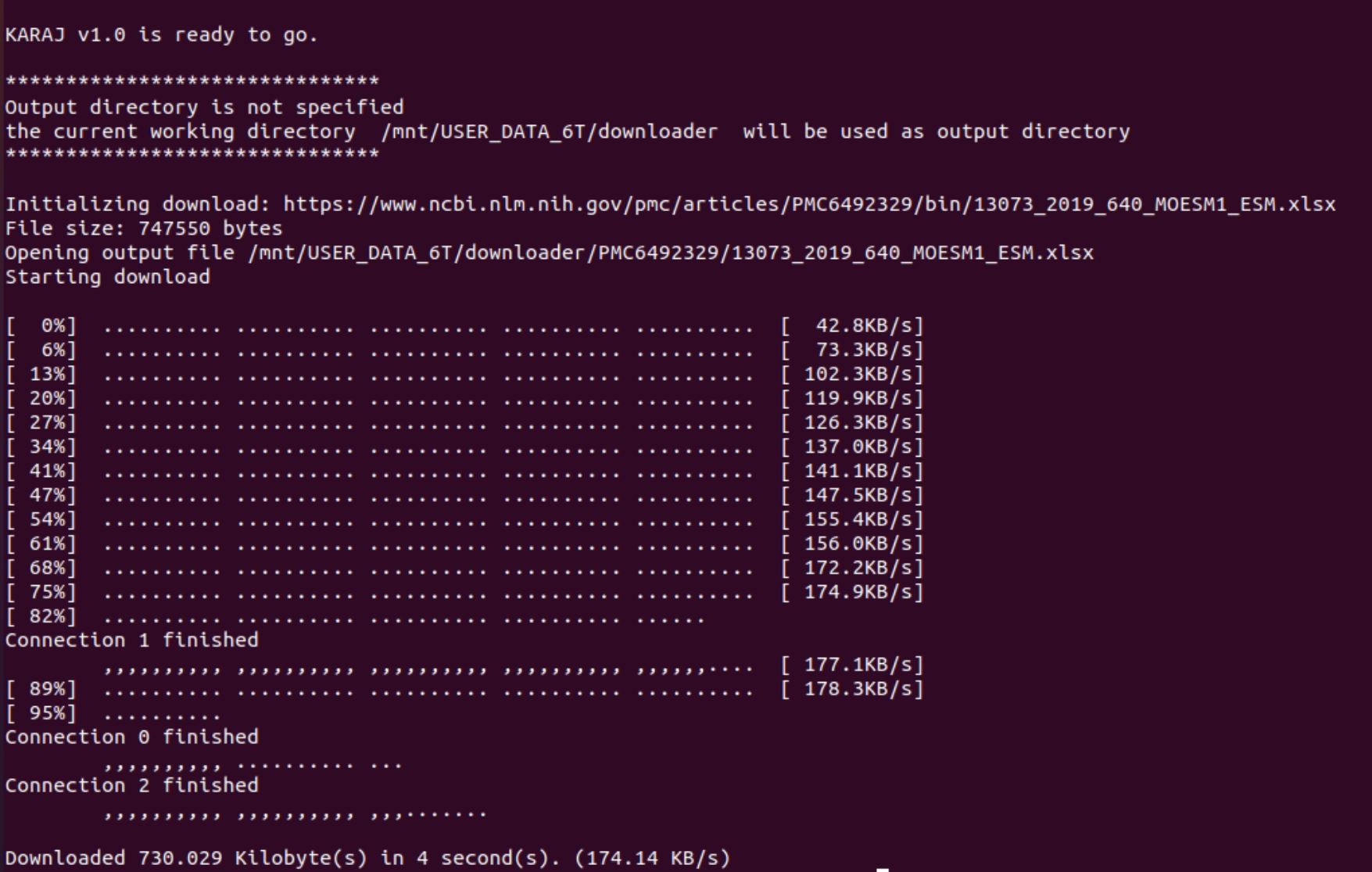

Supplement: Supplementary file 1 [file ijms-23-14418-s001.zip › Supplementary_Figure S3.JPG]

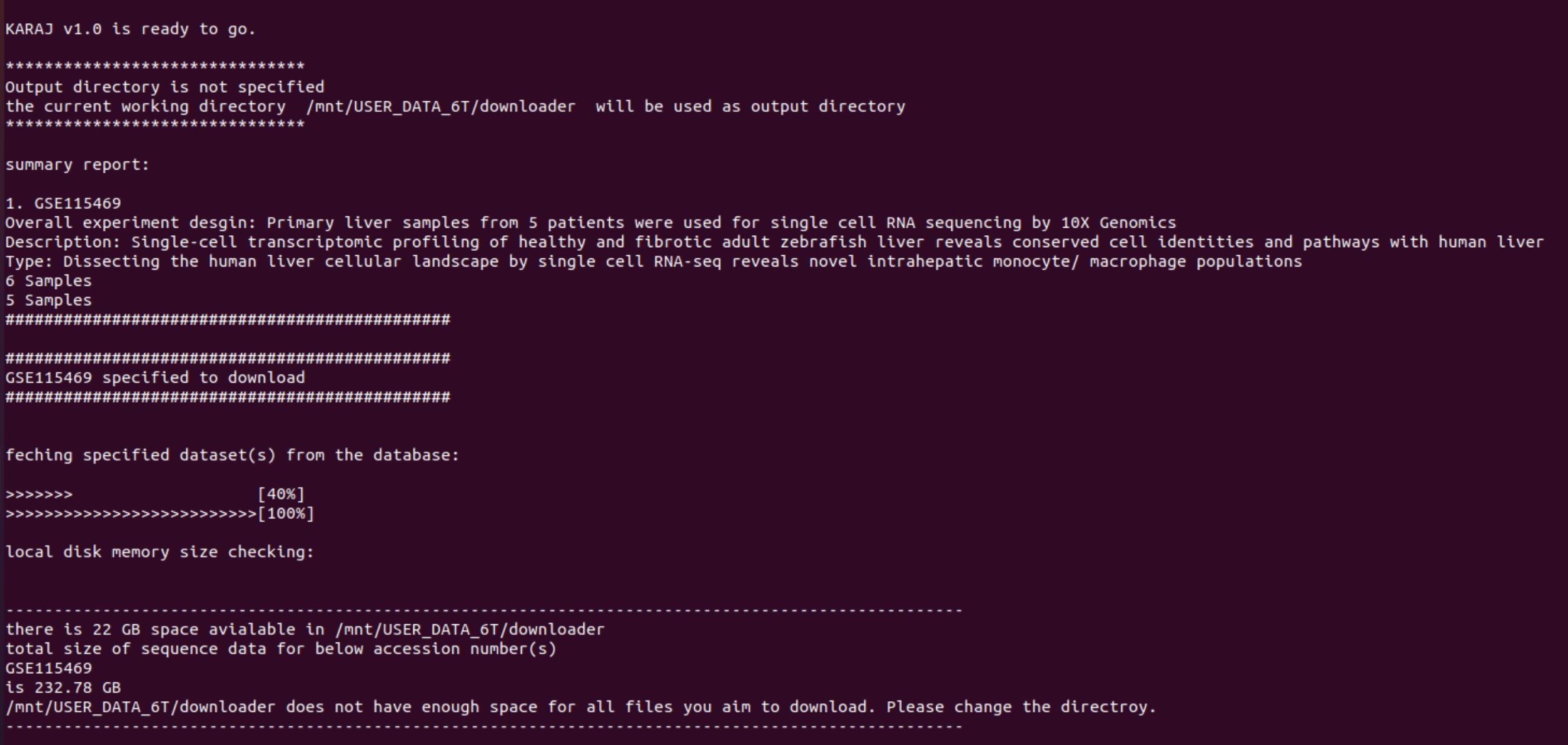

Supplement: Supplementary file 1 [file ijms-23-14418-s001.zip › Supplementary_Figure S4.JPG]
